# DX5^+^NKT cells display phenotypical and functional differences between spleen and liver as well as NK1.1^-^Balb/c and NK1.1^+ ^C57Bl/6 mice

**DOI:** 10.1186/1471-2172-12-26

**Published:** 2011-04-29

**Authors:** Jens M Werner, Elisabeth Busl, Stefan A Farkas, Hans J Schlitt, Edward K Geissler, Matthias Hornung

**Affiliations:** 1 Department of Surgery, University Hospital Regensburg, Franz-Josef-Strauss-Allee 11, 93053 Regensburg, Germany

## Abstract

**Background:**

Natural killer T cells represent a linkage between innate and adaptive immunity. They are a heterogeneous population of specialized T lymphocytes composed of different subsets. DX5^+^NKT cells are characterized by expression of the NK cell marker DX5 in the context of CD3. However, little is known about the phenotype and functional capacity of this unique cell population. Therefore, we investigated the expression of several T cell and NK cell markers, as well as functional parameters in spleen and liver subsets of DX5^+^NKT cells in NK1.1^- ^Balb/c mice and compared our findings to NK1.1^+ ^C57Bl/6 mice.

**Results:**

In the spleen 34% of DX5^+^NKT cells expressed CD62L and they up-regulated the functional receptors CD154 as well as CD178 upon activation. In contrast, only a few liver DX5^+^NKT cells expressed CD62L, and they did not up-regulate CD154 upon activation. A further difference between spleen and liver subsets was observed in cytokine production. Spleen DX5^+^NKT cells produced more Th1 cytokines including IL-2, IFN-γ and TNF-α, while liver DX5^+^NKT cells secreted more Th2 cytokines (e.g. IL-4) and even the Th17 cytokine, IL-17a. Furthermore, we found inter-strain differences. In NK1.1^+ ^C57Bl/6 mice DX5^+^NKT cells represented a distinct T cell population expressing less CD4 and more CD8. Accordingly, these cells showed a CD178 and Th2-type functional capacity upon activation.

**Conclusion:**

These results show that DX5^+^NKT cells are a heterogeneous population, depending on the dedicated organ and mouse strain, that has diverse functional capacity.

## Background

Natural killer T (NKT) cells represent a small but important subset of T lymphocytes with characteristics of both T and NK cells. They have potent immunoregulatory function that reportedly can promote cell-mediated immunity to tumors and infectious organisms and, paradoxically, suppress cell-mediated immunity associated with autoimmune disease and allograft rejection [1]. In mice, these cells express NK cell markers such as NK1.1 and CD94, as well as T-cell receptors (TCR) α/β with a restricted repertoire [2,3]. The invariant T cell receptor α chain Vα14-Jα18 with a conserved CDR3 region is associated with Vβ8.2, Vβ7 or Vβ2 gene segments [3,4].

In contrast to conventional T-lymphocytes, the TCR of NKT cells does not interact with antigens presented by classical major histocompatibility complex (MHC)-encoded class I or II molecules. Instead, their TCR recognizes glycolipids presented by CD1d, which is a MHC class-I-like glycoprotein that belongs to a group of CD1 molecules associated with β2-microglobulin [5-7]. CD1d is known to present lipids including glycosylceramides and glycosylphosphatidylinositol [8,9]. Activation via CD1d initiates the production of both Th1 (IFN) and Th2 cytokines (IL-4, IL-5, IL-13) [10], and increases the cytolytic activity of NKT cells [11].

NKT cells do not represent a homogeneous population. So far, three types of NKT cells have been described. First, there is an invariant Vα14-NKT cell (iVα14-NKT) also called a type I NKT cell or iNKT cell. This group can be further differentiated into CD4^+ ^single positive and CD4^-^CD8^- ^double-negative variants. Second, a population of CD1d-reactive NKT cells expressing diverse non-Vα14TCRs, referred to as type II NKT cells, has also been characterized. A third category has been termed NKT-like cells, which are CD1d-independent and express diverse TCRs [2].

Despite many years of NKT cell research, controversy remains about defining these cells. The expression of several surrogate markers, such as NK1.1 in C57Bl/6 mice and CD161 in humans, co-expressed with the TCR_α/β_, have been frequently used for NKT cell identification [2]. There are also NK1.1^+ ^T cells which do not express the semiinvariant Vα14-Jα18 T cell receptor and are not CD1d-dependent, excluding their consideration as NKT cells.

A common marker for NKT cells in NK1.1^- ^mice strains is the antibody DX5, which recognizes the α_2_-integrin CD49b [12]. DX5 was initially characterized as a marker for NK cells [13] and more recently DX5 co-expressing CD3^+ ^lymphocytes have been described [14]. Several studies, including some from our group, revealed evidence for immunoregulatory properties of these cells [15-18]. DX5^+^NKT cells produce Th1 and Th2 cytokines after stimulation like other NKT cells and seem to play a central role in anti-tumor immunity [13,19]. Recently published studies suggest an immune modulatory function of DX5^+^NKT cells after bone marrow and solid organ transplantation [20,21]. Although all these studies describe typical characteristics of NKT cells, Pellecci et al. showed that DX5^+ ^T cell numbers were normal in CD1d^-/- ^and even in TCR Jα18^-/- ^mice [22]. Therefore, it is more likely that DX5^+^NKT cells belong to the third group of NKT-like cells [22,23].

In the present study we have further characterized DX5^+^CD3^+ ^T cells referred to as DX5^+^NKT cells in NK1.1^- ^(Balb/c) and NK1.1^+ ^(C57Bl/6) mice. For this purpose DX5^+^NKT cells were isolated from spleen and liver and several T and NK cell surface markers as well as maturation and activation markers were studied by flow cytometry. Distinct differences could be found between both subsets and between mouse strains.

## Results

### T cell marker expression is different between spleen and liver DX5^+^NKT cells

**Figure 1:**
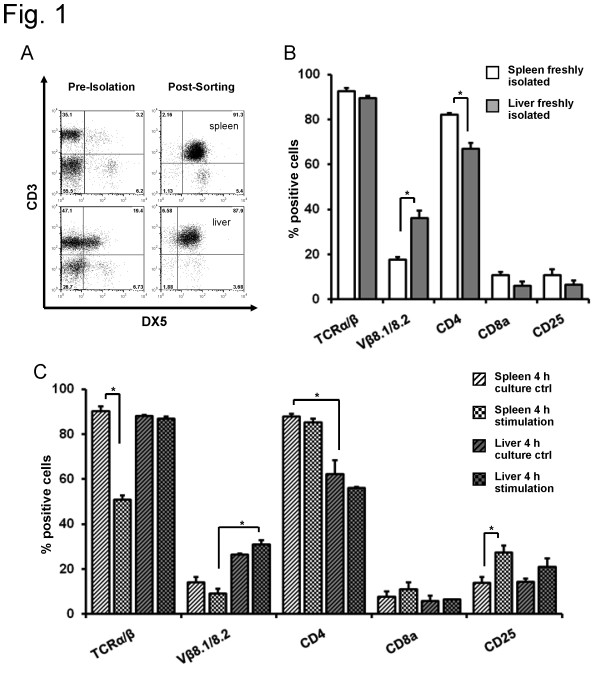
**FACS analysis of T cell marker from spleen and liver CD3^+^/DX5^+^NKT cells in Balb/c mice**. DX5^+^NKT cells were isolated from spleen and liver of Balb/c mice using MACS and FACS-Sorting. Representative dot plot (A). Expression of different T cell markers revealed distinct subsets in Balb/c mice. In the spleen, more DX5^+^NKT cells appeared CD4^+^, in the liver, they expressed more Vβ8.1/8.2 (B). Upon 4 h activation with anti-CD3 and anti-CD28, spleen DX5^+^NKT cells displayed a TCR_α/β _down-regulation, whereas liver DX5+NKT cells did not (C). Results are given as mean + SEM. Experiments were repeated at least three times (* p < 0.05).

Lymphocytes were isolated from spleen and liver of Balb/c mice by MACS and FACS sorting (Figure 1A). First, expression of different T cell markers were analyzed to evaluate which subtype of NKT cells the DX5^+^NKT subset belong to. In the spleen of Balb/c mice, 93 ± 1.4% of freshly isolated DX5^+^NKT cells expressed TCR_α/β_, with 18 ± 1.2% of cells expressing Vβ8.1/8.2 (Figure 1B). Surprisingly, most DX5^+^NKT cells expressed CD4 (82 ± 0.85%), with 11 ± 2% expressing CD8a and CD25. Interestingly, freshly isolated liver DX5^+^NKT cells displayed a different receptor pattern. These cells expressed more Vβ8.1/8.2 (36 ± 3.4%, P = 0.0159) and less CD4 (67 ± 2.6%, P = 0.0079).

Thus far, surface receptor expression was only examined in freshly isolated cells. Since DX5^+^NKT cells display their immunoregulating function especially upon activation [22], expression patterns after stimulation were analyzed. For this purpose, splenic and hepatic mononuclear cells were stimulated for 4 hours with anti-CD3 and anti-CD28 antibodies. Activation of spleen DX5^+^NKT cells from Balb/c mice (Figure 1C) resulted in a down-regulation of TCR_α/β _(90 ± 2% vs. 51 ± 2%, P = 0.004) and an up-regulation of CD25 (14 ± 2.6% vs. 27 ± 3%, P = 0.0411). However, stimulation had little effect on liver derived cells of Balb/c mice. DX5^+^NKT cells revealed an expression pattern similar to that of freshly isolated cells: there was no down-regulation of TCR_α/β _in liver derived DX5^+^NKT cells upon stimulation.

### NK cell and activatory markers are expressed differently in spleen and liver DX5^+^NKT cells

**Figure 2:**
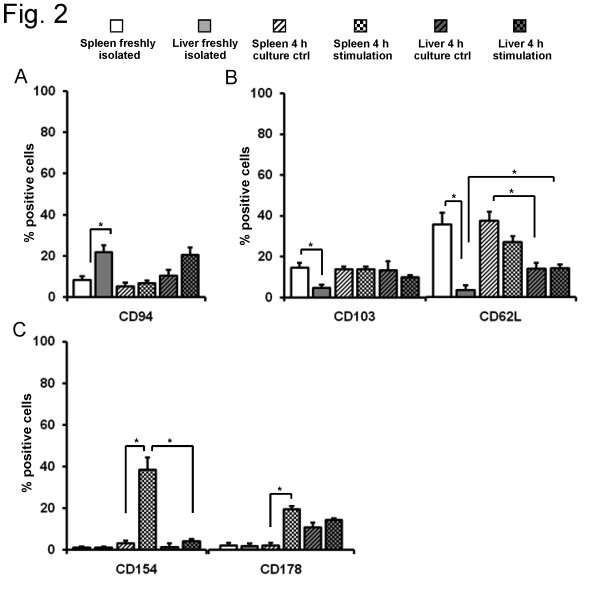
**Activating markers are expressed differently in spleen and liver DX5^+^NKT cells**. Detection of NK cell markers confirmed differences between spleen and liver subsets of DX5^+^NKT cells in NK1.1^-^Balb/c mice. CD94 expression was higher in liver DX5^+^NKT cells (A). Furthermore, the liver subset revealed less expression of maturation marker CD103 and CD62L compared to spleen (B). The activating markers CD154 and CD178 were in both freshly-isolated cell subsets only little expressed (C). Upon stimulation spleen DX5^+^NKT cells displayed an up-regulation of CD154, whereas the liver subset did not. Results are given as mean + SEM. Experiments were repeated at least three times (* p < 0.05).

To further characterize the differences between spleen and liver DX5^+^NKT cells, specific antibodies were used to detect different NK cell types, as well as maturation and activation markers by flow cytometry. In the spleen of Balb/c mice (Figure 2A) the NK cell marker CD94 was expressed on 8 ± 1.8% of DX5^+^NKT cells. Again, DX5^+^NKT cells in the liver displayed differences. CD94 was expressed on significantly more cells than in the spleen (22 ± 3.2%, P = 0.0095). After 4 h stimulation, no significant change in the NK cell receptor pattern was observed in splenic or hepatic DX5^+^NKT cells. As expected, the NK cell marker NK1.1 was expressed only on very few splenic (0.5 ± 0.3%) and hepatic (0.8 ± 0.6%) DX5^+^NKT cells (data not shown).

We also analyzed if the distinct phenotype of spleen and liver DX5^+^NKT cells was based on differences in the maturation status. Therefore, freshly isolated cells were stained with anti-CD103 and anti-CD62L antibodies (Figure 2B). Spleen DX5^+^NKT cells were positive for CD103 in 15 ± 2.4% of cells. In contrast, freshly isolated liver DX5^+^NKT cells expressed this maturation marker less frequently (4.5 ± 1.7%, P = 0.014). Freshly isolated spleen DX5^+^NKT cells were even more frequently positive for the maturation marker CD62L (34 ± 5.5%) compared to liver DX5^+^NKT cells (3 ± 2.2%, P = 0.0079). Upon 4 h stimulation, liver DX5^+^NKT cells displayed an increase in CD62L expression (14 ± 1.7%, P = 0.0317), but still significantly less than the spleen subset (36 ± 4%, P = 0.0028).

Next, differences in activation markers were evaluated. In freshly isolated DX5^+^NKT cells the activation marker CD38 was expressed on approximately 80% of splenic and hepatic cells and displayed no significant change upon stimulation (data not shown). In contrast, upon 4 h stimulation, there was a strong up-regulation of the activating marker CD154 (3 ± 2.2% vs. 39 ± 5.8%, P = 0.0238) in spleen DX5^+^NKT cells (Figure 2C). However, liver DX5^+^NKT cells did not display a similar up-regulation of CD154 (4 ± 1%, P = 0.0498). Another activating marker, CD178, was also not significantly expressed on freshly isolated DX5^+^NKT cells, but upon 4 h stimulation, there was an up-regulation of CD178 in the spleen subset (2 ± 1.3% vs. 19 ± 1.4%, P = 0.0087).

### Spleen and liver DX5^+^NKT cells display a distinct cytokine secretion pattern in Balb/c mice

Results thus far suggest distinct phenotypic differences between spleen and liver DX5^+^NKT cells. Next, the production of Th1 and Th2 cytokines were compared to further assess functional differences between these subsets. Lymphocytes were isolated from spleen and liver of Balb/c mice and cultured for 4, 24 or 48 h in the presence of anti-CD3 and anti-CD28 antibodies. The cells were additionally incubated with PMA, ionomycin and GolgiPlug for intracellular cytokine staining.

**Figure 3:**
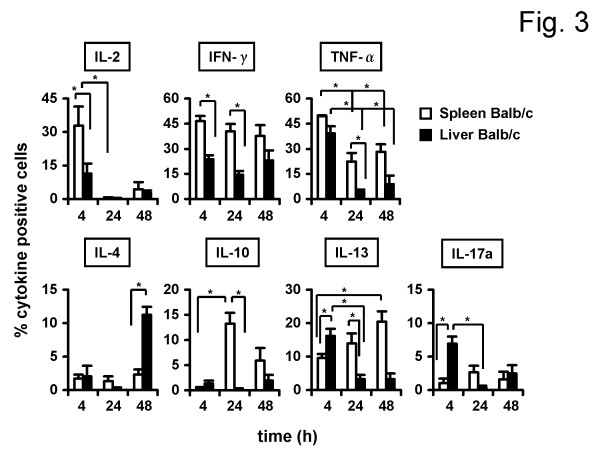
**Spleen and liver DX5^+^NKT cells display a distinct cytokine secretion pattern in Balb/c mice**. Isolated DX5^+^NKT cells were stimulated with antibodies against CD3 and CD28 in the presence of IL-2 for 4, 24 and 48 h, respectively. Control cells were cultured simultaneously without stimulation. Values were subtracted as background. FACS analysis revealed again distinct differences between spleen and liver subsets of DX5^+^NKT cells. Results are given as mean + SEM. Experiments were repeated at least three times (* p < 0.05).

As shown in Figure 3, we first stained for Th1 cytokines such as IL-2, IFN-γ and TNFα. After 4h stimulation, 33 ± 8% of spleen DX5^+^NKT cells produced IL-2. After 24 h, the number of IL-2-producing cells decreased (0.3 ± 0.1%, P = 0.0238). As expected, liver DX5^+^NKT cells showed a distinct Th1 cytokine profile. After 4 h, lower cell numbers produced IL-2 (11 ± 4.4%, P = 0.049) compared to spleen cells. Spleen DX5^+^NKT cells were also positive for IFN-γ after 4 h (46 ± 3.1%), 24 h (40 *± *4.6%) and 48 h (38 ± 6.6%). In contrast, liver DX5^+^NKT cells produced less frequently IFN-γ after 4 h (24 ± 2.4%, P = 0.0079) and 24 h (14.5 ± 2.2%, P = 0.0095). After 4 h splenic DX5^+^NKT cells produced also TNFα (49 ± 0.6%). However, the number of TNFα-producing cells decreased after 24 h (22 ± 5.1%, P = 0.0358). Liver DX5^+^NKT cells again displayed differences; after 4 h they produced slightly less TNFα compared to the spleen subset (39 ± 4.1%) and, after 24 h, the number of TNFα-producing cells decreased even more (4 ± 1.2%, P = 0.0159).

Next, Th2 cytokines such as IL-4, IL-6, IL-10 and IL-13 were analyzed. Only very few spleen DX5^+^NKT cells produced IL-4 after 4, 24 and 48 h. However, the number of IL-4 positive liver DX5^+^NKT cells increased after 48 h (11 ± 1.2%, P = 0.0286). Neither spleen nor liver DX5^+^NKT cells were significantly positive for IL-6 at any time point (data not shown). Spleen DX5^+^NKT cells showed a significant increase in the frequency of IL-10-producing cells after 24 h (13 ± 2.1%) compared to 4 h (0.4 ± 0.2%, P = 0.0187), and compared to liver DX5^+^NKT cells after 24 h stimulation (1 ± 0.6%, P = 0.0357). In the spleen the number of IL-13-producing DX5^+^NKT cells significantly increased after 48 h compared to 4 h (20 ± 3% vs. 10 ± 1.2%, P = 0.0061), whereas in liver the number of IL-13 positive DX5^+^NKT cells peaked after 4 h compared to 24 h (16 ± 2.1% vs. 3 ± 1.2%, P = 0.0286). Furthermore, we tested for IL-17a as a Th17-type cytokine. Liver DX5^+^NKT cells after 4 h more frequently produced IL-17a (7 ± 1%) compared to the splenic-derived cells (1 ± 0.7%, P = 0.0061); after 24 h, the frequency of liver-derived IL-17a-producing DX5^+^NKT cells decreased (0.3 ± 0.3%, P = 0.286).

### NK1.1^+^C57Bl/6 mice reveal distinct surface marker expression on DX5^+^NKT cells

**Figure 4:**
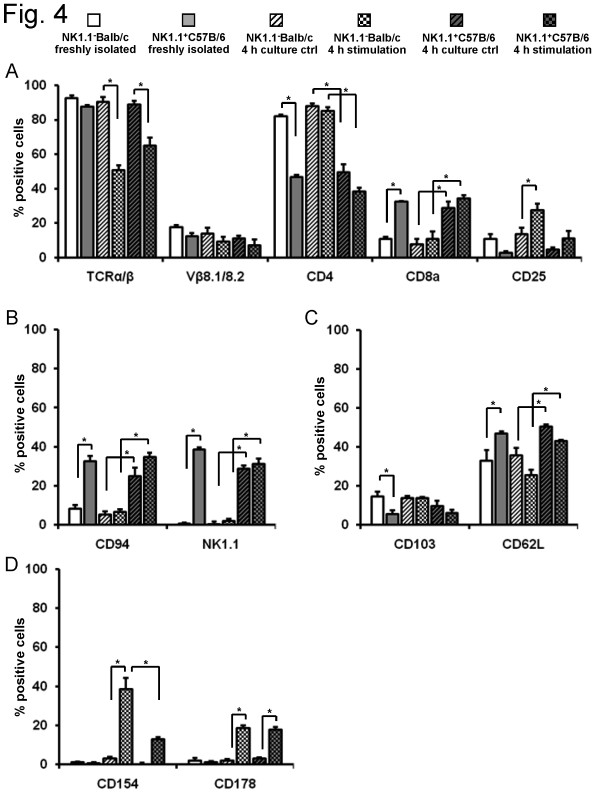
**FACS-Analysis of spleen DX5^+^NKT cells revealed interstrain differences in several phenotypical markers between NK1.1^-^Balb/c and NK1.1^+^C57Bl/6 mice**. In the NK1.1^+ ^mice strain spleen DX5^+^NKT cells expressed less CD4 but more CD8 (A). Upon activation the TCR_α/β _down-regulation was confirmed. In addition to NK.1.1 more spleen DX5^+^NKT cells in C57Bl/6 mice were positive for CD94 (B). Maturation marker CD62L was slightly higher expressed in freshly-isolated spleen DX5^+^NKT cells in C57Bl/6 mice compared to Balb/c, whereas CD103 was less (C). Functional marker CD154 and CD178 were only little expressed in freshly-isolated spleen DX5^+^NKT cells in both mice strains (D). Upon stimulation spleen DX5^+^NKT cells in NK1.1^+ ^C57Bl/6 mice displayed less up-regulation of CD154 compared to Balb/c. Results are given as mean + SEM. Experiments were repeated at least three times (* p < 0.05).

Data thus far were obtained using a NK1.1^- ^mouse strain. These findings were compared to a second mouse strain known to express NK1.1, C57Bl/6 mice.. Several T cell markers were analyzed. A down-regulation of TCR_α/β _(65 ± 4.8% vs. 89 ± 2.1%, P = 0.01) was also observed upon activation of spleen DX5^+^NKT cells from C57Bl/6 mice (Figure 4A). Compared to Balb/c, fewer freshly isolated spleen DX5^+^NKT cells expressed CD4 (82 ± 1% vs. 47 ± 1.1%, P = 0.0043), but more expressed CD8a (11 ± 1.6% vs. 32 ± 0.8%, P = 0.0043). NK cell markers showed significant differences between both mouse strains (Figure 4B). In C57Bl/6 mice 33 ± 2.8% of freshly isolated spleen DX5^+^NKT cells were stained for CD94 (P = 0.0095) and 39 ± 1% for NK1.1 (P = 0.0022). Compared to Balb/c mice (Figure 4C), fewer spleen DX5^+^NKT cells displayed the maturation marker CD103 (15 ± 1.9% vs. 6 ± 1.6%, P = 0.0030), but more expressed CD62L (33 ± 5.5% vs. 47 ± 1%, P = 0.0047). In terms of activating markers, both mouse strains showed little expression of CD154 or CD178 in freshly isolated DX5^+^NKT cells (Figure 4D). However, upon activation, the up-regulation of CD154 (39 ± 5.8% vs. 13 ± 1.2%, P = 0.0012) was less distinctive in C57Bl/6 mice, whereas CD178 expression was similar to Balb/c mice (19 ± 1.4% vs. 18 ± 1.2%).

### Cytokine production of spleen DX5^+^NKT cells is different in NK1.1^-^Balb/c and NK1.1^+ ^C57Bl/6 mice

**Figure 5:**
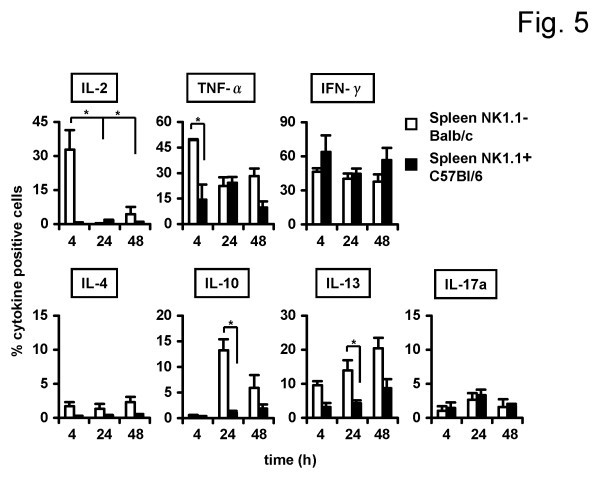
**Cytokine production by spleen DX5^+^NKT cells is different in NK1.1^-^Balb/c compared to the NK1.1^+ ^mice strain C57Bl/6**. Isolated DX5^+^NKT cells were stimulated with antibodies against CD3 and CD28 for 4, 24 and 48 h. FACS-Analysis revealed a Th1 sided cytokine profile for spleen DX5^+^NKT cells of NK1.1^+^C57Bl/6 mice, whereas spleen DX5^+^NKT cells of NK1.1^-^Balb/c mice displayed a pattern with Th1 and Th2 cytokines. Results are given as mean + SEM. Experiments were repeated at least three times (* p < 0.05).

Results have already shown distinct phenotypic differences in DX5^+^NKT cells between NK1.1^- ^and NK1.1^+ ^mice strains. Next, cytokine production was compared to further assess potential functional differences between these subsets. Lymphocytes were isolated from spleens of Balb/c and C57Bl/6 mice and cultured for 4, 24 or 48 h. As shown in Figure 5 spleen DX5^+^NKT cells from the NK1.1^+ ^mouse strain displayed a distinct Th1 cytokine profile. Compared to NK1.1^-^Balb/c mice, DX5^+^NKT cells less often produced IL-2 (33 ± 8.6% vs. 1 ± 0.5%, P = 0.0357) and TNF-α (49 ± 0.6% vs. 14 ± 8.8%, P = 0.0167) after 4h; however, production of IFN-γ was similar. In terms of Th2 cytokines, there was also no significant IL-4 production in spleen DX5^+^NKT cells from the NK1.1^+ ^mouse strain. There were some differences in Th2 cytokine production between NK1.1^- ^and NK1.1^+ ^mice. After 24 h, spleen DX5^+^NKT cells in NK1.1^+ ^C57Bl/6 mice less frequently produced IL-10 (13 ± 2.1% vs. 1 ± 0.3%, P = 0.0357) and IL-13 (14 ± 2.9 vs. 4 ± 0.9%, P = 0.0381). The number of splenic-derived IL-17a-producing DX5^+^NKT cells was similar in both mouse strains. Only a few spleen DX5^+^NKT cells produced IL17a after 4 h and 24 h.

## Discussion

All freshly isolated DX5^+^NKT cells from NK1.1 deficient Balb/c mice were positive for TCR_α/β_, as expected for type III NKT cells [2,3,24]. Most of them additionally expressed CD4, and a few expressed CD8a and CD25. As mentioned in other reports for spleen and thymus [25], a difference between spleen and liver subsets was observed. Liver DX5^+^NKT cells expressed less CD4 and CD62L. Since expression of CD62L is associated with naivety or homing to peripheral lymph nodes in iNKT-cells [26], and CD4 becomes down-regulated after repeated activation [27], this suggests a higher maturational status of liver DX5^+^NKT cells in Balb/c mice. This is supported by the finding that Vβ8.2-containing V*α*14*i *TCRs in NKT cells have a higher antigen affinity than those containing Vβ7 or Vβ2 [28,29]. Consistent with the idea of a negative selection, liver DX5^+^NKT cells displayed a higher expression of Vβ8.1/8.2.

Upon activation, a down-regulation of TCR_α/β _was observed in spleen DX5^+^NKT cells in Balb/c mice. A decrease of TCR_α/β_-expression was also reported for NK1.1^+^NKT cells [30]. Due to a lack of detection, a rapid apoptotic death of this cell was initially claimed upon stimulation [30]. More recently TCR_α/β_-down-regulation in spleen and liver NK1.1^+^NKT cells of C57Bl/6 mice was discovered to be the reason for this assumed cell disappearance [31,32]. However, our study does not confirm this finding for liver DX5^+^NKT cells in Balb/c mice, supporting evidence that there are distinct subsets of DX5^+^NKT cells in the spleen and liver. CD154 expression was also increased upon stimulation. As the ligand of CD40, CD154 represents a co-stimulatory molecule and is expressed by activated and regulatory T cells [33,34]. Furthermore, CD178, the ligand of Fas responsible for the extrinsic induction of apoptosis (e.g. expressed on CD8^+ ^cytotoxic T cells) was up-regulated [35]. This up-regulation was more evident in spleen than in liver DX5^+^NKT cells and is also reported for NK1.1^+^NKT cells [36]. Taken together, liver DX5^+^NKT cells are more mature and display a different activating phenotype upon stimulation compared to the spleen subset.

Since NKT cells are supposed to link innate and adaptive immunity by rapid secretion of cytokines, including IFNγ, TNFα, IL-4, IL-10, IL-13, GM-CSF and IL-2 [24,37,38], we further assessed differences between spleen and liver DX5^+^NKT cells on a functional basis. Fewer liver DX5^+^NKT cells in Balb/c mice produced Th1 cytokines such as IL-2, IFN-γ, and TNF-α. In terms of Th2 cytokines, more liver DX5^+^NTK cells produced IL-4, but fewer produced IL-10 compared to the spleen subset. Charbonnier et al. described an induction of IL-10 production in liver CD49b^+^CD4^+ ^T cells by immature dendritic cell vaccination [18]. However, we did not confirm this finding for liver DX5^+^NKT cells in Balb/c mice after anti-CD3/anti-CD28 stimulation. Collectively, the limited Th1 cytokine secretion upon stimulation confirmed the observation that a different activating phenotype of DX5^+^NKT cells exists in the liver of Balb/c mice.

Recently, type I NKT cells have been shown to be capable of producing IL-17, potentially implicating this cell type in inflammatory conditions [39,40]. We showed that a subset of DX5^+^NKT cells in the liver of Balb/c mice has to be taken into account as an IL-17 producing cell. These results are consistent with our result regarding a more mature phenotype of liver-derived DX5^+^NKT cells, with a different phenotypical profile upon stimulation compared to the spleen subset.

In NK1.1^+ ^C57Bl/6 mice fewer spleen DX5^+^NKT cells revealed expression of CD4, but more of CD8a. These findings are consistent with data from Pellici et al., who observed similar differences in C57Bl/6 mice [22]. In accordance with Conzalez et al., 40-50% of DX5^+^NKT cells express NK1.1 [15]. Taking these results together with the higher CD4 expression in NK1.1-deficient Balb/c mice, we suggest that these mice compensate for a reduced number of NK.1.1^+^NKT cells with a higher prevalence of CD4^+^DX5^+^NKT cells. In contrast to results from Gonzalez et al. [15], about one-third of these cells in the spleen of C57Bl/6 mice, and 11% in Balb/c mice, were CD8^+^.

Upon activation a down-regulation of TCR_α/β _was observed in spleen DX5^+^NKT cells in Balb/c and in C57Bl/6 mice, as it has been reported for NK1.1^+^NKT cells [30]. Furthermore, upon a 4 h stimulation there was a notable up-regulation of CD25 in Balb/c versus C57Bl/6 mice. CD25, the IL-2 receptor α-chain, is proposed to be a phenotypical marker of regulatory T cells [41-43]. The up-regulation we observed especially for spleen DX5^+^NKT cells in Balb/c mice could be either due to activation and increased receptor expression based on the regulatory background of DX5^+^NKT cells or due to a natural selection because of a higher apoptotic resistance in CD4^+^CD25^+ ^T cells [44]. Another marker for activation on regulatory T cells is CD154 [34]. CD154 expression was increased upon stimulation in spleen DX5^+^NKT cells in Balb/c mice, but was significantly less prominent in C57Bl/6 mice. Upon 4 h stimulation, CD178 (ligand of Fas) was similarly up-regulated [35] on splenic DX5^+^NKT cells in both mice strains. However, after 24 h stimulation CD178 expression was decreased in Balb/c versus C57Bl/6 mice, and CD154 was further up-regulated in Balb/c, but down-regulated in C57Bl/6 mice (data not shown). These changes suggest a more regulatory CD4^+^CD25^+ ^phenotype in NK1.1^- ^Balb/c and a more CD8^+ ^and CD178^+ ^driven cytotoxic component in the NK1.1^+ ^C57Bl/6 mouse strain [45].

High IL-2 production by DX5^+^NKT cells in the early phase of activation was observed only in Balb/c mice, further supporting our findings of interstrain differences. Moreover, splenic DX5^+^NKT cells in C57Bl/6 mice displayed a more one-sided Th1 cytokine profile, with mostly IFNγ and TNFα producing cells, which is also described for CD8^+ ^T cells [46]. DX5^+^NKT cells in Balb/c mice displayed a more polyfunctional cytokine profile. In the late phase of stimulation, IFNγ and TNFα production decreased and IL-10 and IL-13 secretion increased. In contrast to liver CD49b^+^CD4^+ ^cells [18], and supporting our data regarding splenic DX5^+^NKT cells, Kassiotis et al. revealed no IL-10 production for splenic CD49b^+^CD4^+ ^T cells in C57Bl/6 mice [47]. Furthermore, Pellicci et al. could not detect any significant IL-10 production in DX5^+^TCR_α/β_^+^CD1d-Tet^+ ^cells in the same mouse strain [22]. Confirming our finding and giving again evidence for a difference between both mouse strains, we showed a significant increase in IL-10 production after 24h stimulation for splenic DX5^+^NKT cells in Balb/c mice. Taken together, these results suggesting a more one-sided Th1 cytokine production from DX5^+^NKT cells in C57Bl/6 mice confirm their more CD8 and CD178 expressing phenotype.

## Conclusions

In conclusion, our data show that distinct subsets of DX5^+^NKT cells exist within NK1.1^- ^Balb/c mice. In the spleen these cells appear more naive with a higher CD62L expression and an up-regulation of CD154 and CD178 upon activation. In contrast, in the liver DX5^+^NKT cells are more mature and display a different regulatory potency upon stimulation. Furthermore, there are remarkable inter-strain differences. In NK1.1^+ ^C57Bl/6 mice, fewer DX5^+^NKT cells are CD4^+ ^and more are CD8^+^, confirming their CD178 and Th1-sided functional behavior upon activation.

## Methods

### Cell harvesting and isolation

All experiments were approved by the institutional animal care committee at the University Hospital Regensburg. Different lymphocytes subsets were purified from splenic or hepatic mononuclear cells isolated from Balb/c mice or C57Bl/6NCrl mice (Charles River Laboratories, Wilmington, MA, USA). If necessary, further isolation was performed by magnetic activated cell sorting (MACS; Miltenyi Biotec, Bergisch Gladbach, Germany) or by FACS (FACSAria I, BD Bioscience, San Jose, CA, USA). Briefly, cell suspension of the spleen was prepared by cutting the tissue into small pieces and gently pressing through a 100 μm wire mesh. For preparation of the liver the portal vein was flushed with 10 ml sodium chloride until the organ became pale. Then the liver was cut into small parts and passed through a 100 μm wire mesh. Hepatic lymphocytes were further isolated by density gradient centrifugation using 80% and 40% Percoll (Biochrom, Berlin, Germany). For further isolation, DX5^+ ^cells were purified using anti-mouse-DX5^+ ^MicroBeads (Miltenyi Biotec). Cells were passed through a MACS-column (type LS) attached to a Midi-MACS-magnet (Miltenyi Biotec). DX5^+ ^cells were labeled with FITC-conjugated anti-mouse CD3 (clone: 17A2, rat IgG2b) and PE-conjugated anti-mouse CD49b (clone: DX5, rat IgM) (all from BD Biosciences) for further CD3^+^DX5^+ ^enrichment by FACS, and are referred to as DX5^+^NKT cells throughout the study.

### Antibodies and flow cytometry

The following reagents were used for cell surface labeling in multiparameter flow cytometric analysis (FACS Calibur, BD Bioscience): FITC or Alexa Fluoar 647-conjugated anti-mouse CD3 (clone: 17A2, rat IgG2b), APC-conjugated anti-mouse-CD4 (clone: GK1.5, rat IgG2b), APC-conjugated anti-mouse-CD8a (clone: 53-6.7, rat IgG2a), PE-conjugated anti-mouse-CD38 (clone: 90, rat IgG2a), PE-conjugated anti-mouse-CD49b (clone: DX5, rat IgM), APC-conjugated anti-mouse-CD62L (clone: MEL-14, rat IgG2a), PE-conjugated anti-mouse-CD103 (clone: M290, rat IgG2a), PE-conjugated anti-mouse-CD178 (Fas-Ligand) (clone: MFL3, hamster IgG1), PE-conjugated anti-mouse-Vβ 8.1, 8.2 TCR (clone: MR5-2, rat IgG2a) all BD Biosciences. APC-conjugated anti-mouse-CD49b (clone: DX5, rat IgM), FITC-conjugated anti-mouse-CD49b (clone: DX5, rat IgM) all obtained from Miltenyi Biotec. FITC-conjugated anti-mouse-CD94 (clone: 18d3, rat IgG2a), APC-conjugated anti-mouse-CD154 (CD40L) (clone: MR1, hamster IgG), APC-conjugated anti-mouse-NK1.1 (clone: PK136, mouse IgG2a) all obtained from eBioscience (San Diego, CA, USA). APC-conjugated anti-mouse-CD25 (clone: PC61 5.3, rat IgG1), FITC-conjugated anti-mouse-TCRα/β (clone: HM 3601, hamster IgG) from Caltag (Towcester, UK).

### Activation of DX5^+^NKT cells

For lymphocyte stimulation, 96 well cell culture plates (Corning costar, Sigma Aldrich) were coated with anti-mouse-CD3e (Clone: 145-2C11, BD Biosciences) at 10 μg/ml and stored overnight at 4°C. Wells were washed twice with PBS. And up to 1 × 10^6 ^isolated lymphocytes from spleen and liver were plated in 200 μl RPMI culture medium (Biochrom). For activation, 5 μg/ml of anti-mouse-CD28 (clone: 37.51, BD Biosciences) and 2000 IU/ml IL-2 (Peprotech, Rocky Hill, NJ, USA) were added. Plates were incubated for the indicated time at 37°C and 5% CO_2_

### Intracellular cytokine staining

4 × 10^5 ^DX5^+^-NKT cells were incubated in 200 μl culture medium in a 96 well plate as mentioned above. Additionally, 50 ng/ml PMA (InvivoGen, San Diego, CA, USA) was added at the beginning, with 750 ng/ml ionomycin (Sigma-Aldrich, St. Louis, USA) being added for the last 4h; 1 μg/ml GolgiPlug (BD Bioscience) was added 2 h before cell harvesting. Culture supernatants were harvested and stored at -20°C for IFNγ ELISA. Cells were fixed in 1ml Fix/Perm (eBioscience) for 60 min at 4°C. After incubation with permeabilization buffer (eBioscience) cells were stained with PE-conjugated anti-mouse-cytokine Abs (IL-2, clone: JES6-5H4/IL-4, clone: BVD4-1D11/IL-6, clone: MP5-20F3/IL-10, clone: JES5-16E3/IFNγ, clone: XMG1.2/TNFα, clone: MP6-XT22) from BD Bioscience and with PE-conjugated anti-mouse-Abs (IL-13, clone: eBio13A/IL-17a, clone: eBio17B7) and FITC-conjugated anti-mouse-IFNγ (clone: XMG1.2) (all eBioscience).

### IFNγ analysis by Enzyme-Linked Immunosorbent Assay

Stimulation of DX5^+^NKT cells was confirmed by IFNγ ELISA. IFNγ concentration from harvested supernatants was analyzed using a commercially available sandwich ELISA kit (BD Bioscience). Tetramethylbenzidine dihydrochloride was used for detection. ELISA readings were determined by OD scanning at 450 nm using an Emax precision microplate reader from Molecular Devices (Downingtown, PA, USA).

### Statistics

All in vitro experiments were repeated at least 3 times and data are presented as the mean value ± SEM. Statistical analyses were performed using either a student's t-test or the Mann-Whitney-U-test. Differences were considered significant at P < 0.05.

## Authors' contributions

JMW carried out FACS analysis, participated in the design of the study, data analysis and the preparation of the manuscript. EB carried out isolation of cells and FACS analysis. SAF participated in the design and coordination of the study and helped to draft the manuscript. HJS participated in the design of the study and revised the manuscript and gave important intellectual content. EKG has made substantial contributions to the conception and the design of the study, to the analysis and interpretation of the data and revised the manuscript. MH conceived of the study, and participated in its design and coordination, analyzed the data and drafted the manuscript. All authors read and approved the final manuscript.
